# Prognostic significance of delirium in patients with heart failure: a systematic review and meta-analysis

**DOI:** 10.3389/fcvm.2023.1217965

**Published:** 2023-08-11

**Authors:** Ziru Niu, Jiamin Zhou, Yongjun Li

**Affiliations:** ^1^ Department of Cardiovascular Medicine, The Second Hospital of HeBei Medical University, Shijiazhuang, China; ^2^ Department of Hematology, The Second Hospital of HeBei Medical University, Shijiazhuang, China

**Keywords:** delirium, heart failure, mortality, hospitalization, meta-analysis

## Abstract

**Background:**

Delirium is a common symptom of heart failure (HF) and is associated with increased mortality, prolonged hospital stays, and heightened medical costs. The impact of delirium on the prognosis of HF patients is currently controversial. Therefore, we conducted a meta-analysis to evaluate the prognostic significance of delirium in HF.

**Methods:**

Relevant articles were systematically searched in PubMed, Cochrane Library, Web of Science, and Embase based on the PRISMA guidelines. Studies that reported mortality and hospitalization-related outcomes in HF patients with or without delirium using raw or adjusted hazard ratio (HR) and odds ratio (OD) were included. Meta-analysis was then performed to evaluate the effect of delirium in HF patients. Outcomes of interest were all-cause mortality and events of the hospitalization.

**Results:**

Of the 1,501 studies identified, 7 eligible studies involving 12,830,390 HF patients (6,322,846 males and 6,507,544 females) were included in the meta-analysis. There were 91,640 patients with delirium (0.71%) and 12,738,750 patients without delirium (99.28%). HF patients with delirium had higher OR for in-hospital mortality (1.95, 95% CI = 1.30–2.91, *P *= 0.135), higher pooled HR for 90-day mortality (2.64, 95% CI = 1.06–1.56, *P = *0.215), higher pooled HR for 1-year mortality (2.08, 95% CI = 1.34–3.22, *P *= 0.004), and higher pooled HR for 30-day readmission rate (4.15, 95% CI = 2.85–6.04, *P* = 0.831) than those without delirium.

**Conclusion:**

Current evidence suggests that combined delirium increases the risk of HF-related mortality and hospitalization-related outcomes in patients with HF. However, more research is needed to assess the impact of delirium on the prognosis of HF patients.

## 1. Introduction

Heart failure (HF) is a complex clinical syndrome characterized by structural or functional impairment in ventricular filling or blood ejection (1). HF has high morbidity and mortality rates worldwide and is the most life-threatening cardiovascular disease, posing huge social and economic burdens in both developed and developing countries (2). HF has been recognized as a global pandemic, affecting approximately 634 million people worldwide (3). A recent study in the US found that the total number of HF deaths has risen from 275,000 cases in 2009 to 310,000 cases in 2014 (4). With the acceleration of population aging and continuous improvement in medical care level, the number of HF patients is also expected to increase. At present, HF is an important global public health problem, and many HF patients have significantly declined quality of life (QoL) (5, 6). In addition, the presence of comorbidities such as delirium makes it difficult for HF patients to communicate normally with medical staff, which increases the difficulty for medical staff to evaluate the patients’ conditions (7).

Delirium is a disorder characterized by dramatic changes in attention, awareness, and cognition, and is caused by an underlying medical condition that is not better explained by another preexisting neurocognitive disorder (8). Delirium is officially defined in the Diagnostic and Statistical Manual of Mental Disorders, fifth edition (DSM-5) (6) as a condition with the following five key features: Disturbance in attention and awareness; the disturbance develops over a short period of time and its severity tends to fluctuate during the course of a day; an additional disturbance in cognition; these mentioned disturbances cannot be better explained by other preexisting neurocognitive disorders and do not occur in severely reduced arousal level such as coma; and there is evidence suggesting the disturbance is a direct result of another medical condition. Several delirium assessment tools can highly improve the diagnostic accuracy and quantification of the severity of delirium, including the confusion assessment method (CAM), Memorial Delirium Assessment Scale (MDAS), and Delirium Rating Scale-R-98 (DRSR-98)8. CAM is the most widely used tool with high sensitivity (94%–100%) and specificity (90%–95%) (9).

Delirium has been shown to be independently associated with multiple adverse outcomes, including increased mortality after hospital discharge, new institutionalization, and dementia (10). Risk factors for delirium include increased age, mental illness, alcohol abuse, cognitive impairment, malnutrition, vision and hearing impairment, and cardiovascular disorders (8, 11–15).

Several studies have shown that delirium is a common symptom of HF patients and is associated with increased mortality, prolonged hospital stays, and heightened medical costs. Delirium seriously affects the health and QoL of patients and imposes a heavy burden on medical resources (16–23). It was reported that HF is an independent risk factor for delirium (24). Uthamalingam et al. found that 17% of decompensated HF patients displayed symptoms of delirium during hospitalization (24). Nevertheless, the diagnosis of delirium in HF patients is often untimely, and current medical care is also insufficient to meet the needs of such patients (8). Specific training on delirium in cardiology is currently lacking (25), and there are no guidelines or clinical pathways specific for delirium in HF patients. This may be attributed to the unclear and still highly-debated effect of delirium on HF prognosis. We aim to systematically synthesize the published evidence on the associations between delirium and the prognosis of HF patients. To our knowledge, no published study has systematically synthesized this evidence.

## 2. Methods

### 2.1. Protocol and registration

The systematic review and meta-analysis were conducted based on the Preferred Reporting Items for Systematic and Meta-analysis (PRISMA) (26) protocols and the Meta-analysis of Observational Studies in Epidemiology checklist. This protocol was registered on PROSPERO (27) on August 8, 2022 (Registration number CRD42022348656).

### 2.2. Search strategy

Relevant articles were searched in PubMed, EMBASE, Cochrane Library (CENTRAL), and Web of Science from inception to June 27, 2022 using different combinations of the MeSH terms “deliration”, “delirium”, “phrenitis”, “Delirium”, “heart failure”, “cardiac failure”, “Myocardial Failure”, “Heart Decompensation”; “Decompensation, Heart”, “heart failure”, and “weak heart”.

### 2.3. Eligibility criteria

The references of the included studies were manually screened by two researchers to further identify relevant studies. Study inclusion criteria: (1) Evaluated morbidity rate of HF patients with delirium; (2) Conducted in humans; (3) Assessed the impact of delirium on short- and long-term mortality rate and hospitalization (hospitalization time is defined as “length of stay at hospital, Days”); (4) Reported mortality rate and hospitalization using original and/or adjusted HR, OR; (5) Reported effect size and its 95% confidence interval (CI) after logistic regression or multivariate cox regression correction; (6) Observational, prospective or retrospective study; (7) Published in a peer-reviewed journal or conference; (8) Written in English.

### 2.4. Data extraction

Literature screening, data extraction, and cross checking were performed independently by two researchers. Any disagreement was resolved by consultation and discussion with a third researcher. The article title was first screened to exclude irrelevant studies, and then the abstracts and full texts were read to identify studies that met the inclusion criteria. Data that were extracted included basic study information (e.g., author information and descriptive data), outcome data (mortality and hospitalization events) and degree of statistical adjustment for any potential confounders used (+ = no adjustment; ++ = adjustment for age, sex, and some standard heart failure risk factors; +++ = adjustment for the preceding cognitive impairment). If both multivariate and univariate analyses are available, data from the multivariate analysis are preferentially extracted.

### 2.5. Risk of bias assessment

The Newcastle Ottawa Scale (NOS) (28) was used to independently assess the risk of bias and cross-check the results. The NOS is an effective method for evaluating the quality of systematic reviews of observational studies. The evaluation includes three domains, namely the selection of research objects (four points), control of confounding factors in the study cohort (two points), and judgment of outcome events (three points). There are eight items on the scale, with a total score of 9.0. A score of 7.0–9.0 indicates high quality, while a score of 4.0–6.0 indicates medium quality (28).

### 2.6. Strategy for data synthesis

The morbidity and prognosis data of HF patients with delirium extracted from each study were statistically analyzed, and meta-analysis was performed using Stata 16.0. Morbidity rate and adjusted hazards ratio (HR)/odds ratio (OR) were pooled. The HR/OR is used to assess the effect of delirium on the risk of death. I^2^ test was used to detect heterogeneity. The *I*^2^ values of 25%, 50%, and 75% represented low, moderate, high heterogeneity, respectively (29). If *I*^2^ is ≥50% (presence of heterogeneity), a random effects model is used; otherwise, a fixed effects model is used. The source of heterogenicity was identified by meta-regression and subgroup analysis.

## 3. Results

### 3.1. General characteristics of included studies

**Figure 1:**
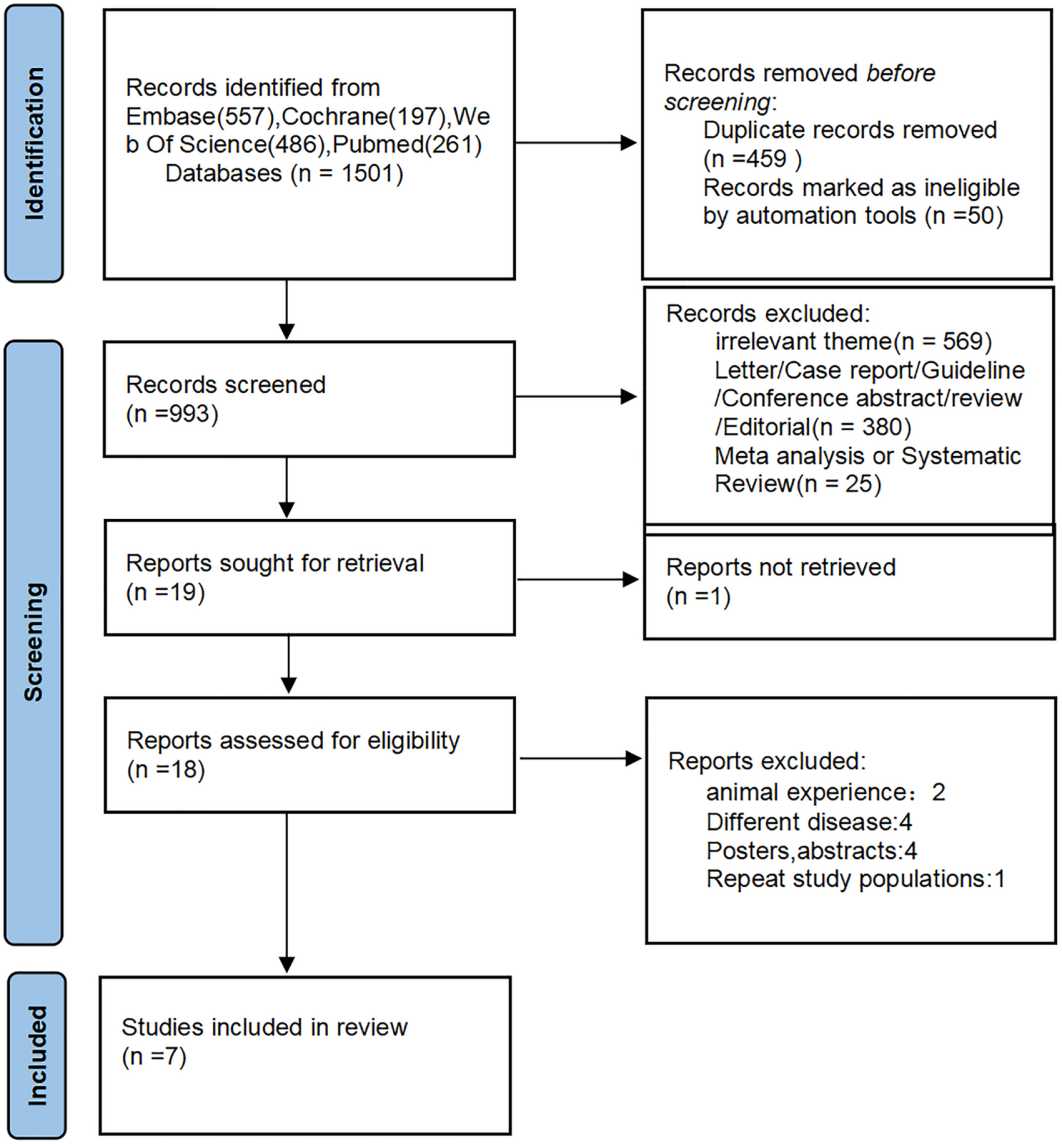
The PRISMA flowchart.

Of the 1,501 articles identified using the MeSH terms, 7 were eligible for inclusion in the meta-analysis (Figure 1). There were four retrospective cohort studies (16, 17, 23) and four prospective cohort studies (19–22) four studies were conducted in the United States (16, 17, 21, 23), two in Japan (19, 20), and one in Spain (22). Three of these studies reported ORs/HRs adjusted for cognitive impairment. The characteristics of the included studies are summarized in Table 1.

**Table 1:** Details of the included studies.

Author	Study type	Country	Type	Sample	Age (year)	Male (%)	Incidence (%)	Degree of adjustment	Diagnostic criteria	Follow-up (day)	Outcomes
Ritchie et al. (16)	Retrospective cohort study	USA	HF	12804794	>18	49.2	0.70%	++	Electronic code	NA	a,b,c
Lafo et al. (17)	Retrospective cohort study	USA	HF	21655	77.01	97	5.92%	++	Electronic code	365	g,h,d,f
Pak et al. (19)	prospective cohort study	Japan	ADHF	132	83	51.5	27.40%	++	DSM-5	90	e
Iwata et al. (20)	Prospective cohort study	Japan	AHF	408	>20	52.46	26.70%	++	CAM-ICU	365	a,f,b
Ayatollahi et al. (21)	Prospective cohort study	USA	DHF	2279	≥65	47.6	23.80%	+++	CAM	30	g
Alberto et al. (22)	Prospective cohort study	Spain	DHF	239	81.7	38.9	14.60%	+++	CAM	365	f
Uthamalingam et al. (23)	Retrospective cohort study	USA	ADHF	883	>65	48	17.10%	+++	CAM	90	a,e,g

HF, heart failure; AHF, acute heart failure; ADHF, acute decompensated heart failure; DHF, decompensated heart failure;77.01 = average age of 77.01(SD = 10.52); 83 = 83 (interquartile range, 75–87) years; 81.7 = 81.7 ± 9.4 years; DSM-5, diagnostic and statistical manual of mental disorders, fifth edition; CAM, confusion assessment method; CAM-ICU, confusion assessment method for intensive care unit; degree of adjustment: + = no adjustment; ++ = adjustment for age, sex, and some standard heart failure risk factors; +++ = adjustment for the the preceding cognitive impairment; a = in-hospital mortality; b = length of stay; c = total hospital cost; d = 30 day mortality; e = 90 day mortality; f = 365 day mortality; g = 30 day readmission; h = 365 day readmission.

The quality of the 7 included articles (16, 17, 19–23) was evaluated by NOS. The NOS scores of all involved studies were above six, which indicates a low risk of bias (Table 2).

**Table 2:** The Newcastle-Ottawa scale.

Study	Cohort selection				Comparability	Outcome			Total score
	A	B	C	D	E	F	G	H	
Ritchie et al. (16)	1	1	1	1	2	1	1	1	9
Lafo et al. (17)	0	0	1	1	2	1	1	1	7
Pak et al. (19)	1	1	1	1	2	1	0	1	8
Iwata et al. (20)	1	1	1	1	2	1	1	1	9
Ayatollahi et al. (21)	0	0	1	1	2	1	1	1	7
Alberto et al. (22)	1	1	1	1	2	1	1	1	9
Uthamalingam et al. (23)	1	1	1	1	2	1	0	1	8

A, Representativeness of the exposed cohort; B, Representativeness of the non-exposed cohort; C, Ascertainment of exposure; D, Outcome of interest was not present at start of study; E, Comparability of the exposed and non-exposed cohorts; F, Assessment of outcome; G, Was the follow-up long enough for outcomes to occur; H, Adequacy of follow-up.

### 3.2. Identification of delirium

There are several delirium assessment tools that can quantify the severity of delirium, including the Confusion Assessment Method (CAM), Memorial Delirium Assessment Scale (MDAS), and Delirium Rating Scale R-98 (DRS-R-98) (30, 31). CAM-ICU, an adaptation of CAM, is often used for patients with critical illness, especially those who cannot communicate verbally (32).

Only one study (19) used the Diagnostic and Statistical Manual of Mental Disorders, fifth edition (DSM-5) (6) to diagnose delirium. Six studies (17, 20–23) reported the use of delirium assessment tools for delirium diagnosis, such as CAM or CAM-ICU. One study (16) used the Electronic Code to identify delirium patients.

### 3.3. Description of the study populations

The 7 included studies involved 12,830,390 HF patients, among which 6,322,846 were males, 6,507,544 were females, 91,640 had delirium (0.71%), and 12,738,750 did not have delirium (99.28%). One studies (20) reported cases of acute HF, and 4 studies (17, 21–23) reported cases of decompensated HF. The remaining two studies (16, 17) did not report the characteristics of their HF patients (Table 1).

Three studies reported the mean age (standard deviation, SD) at the follow-up to be between 77.01 (SD = 10.52) and 83 (interquartile range, 75–87) years. Three studies (16, 19, 21) included only elderly subjects who were >65 years old, and two studies (16, 20) included patients who were 18 and 20 years of age.

All 7 studies reported the sex distribution of the patients, and the percentage of male patients was 38.9% (22) to 97% (17).

### 3.4. Mortality

**Figure 2:**
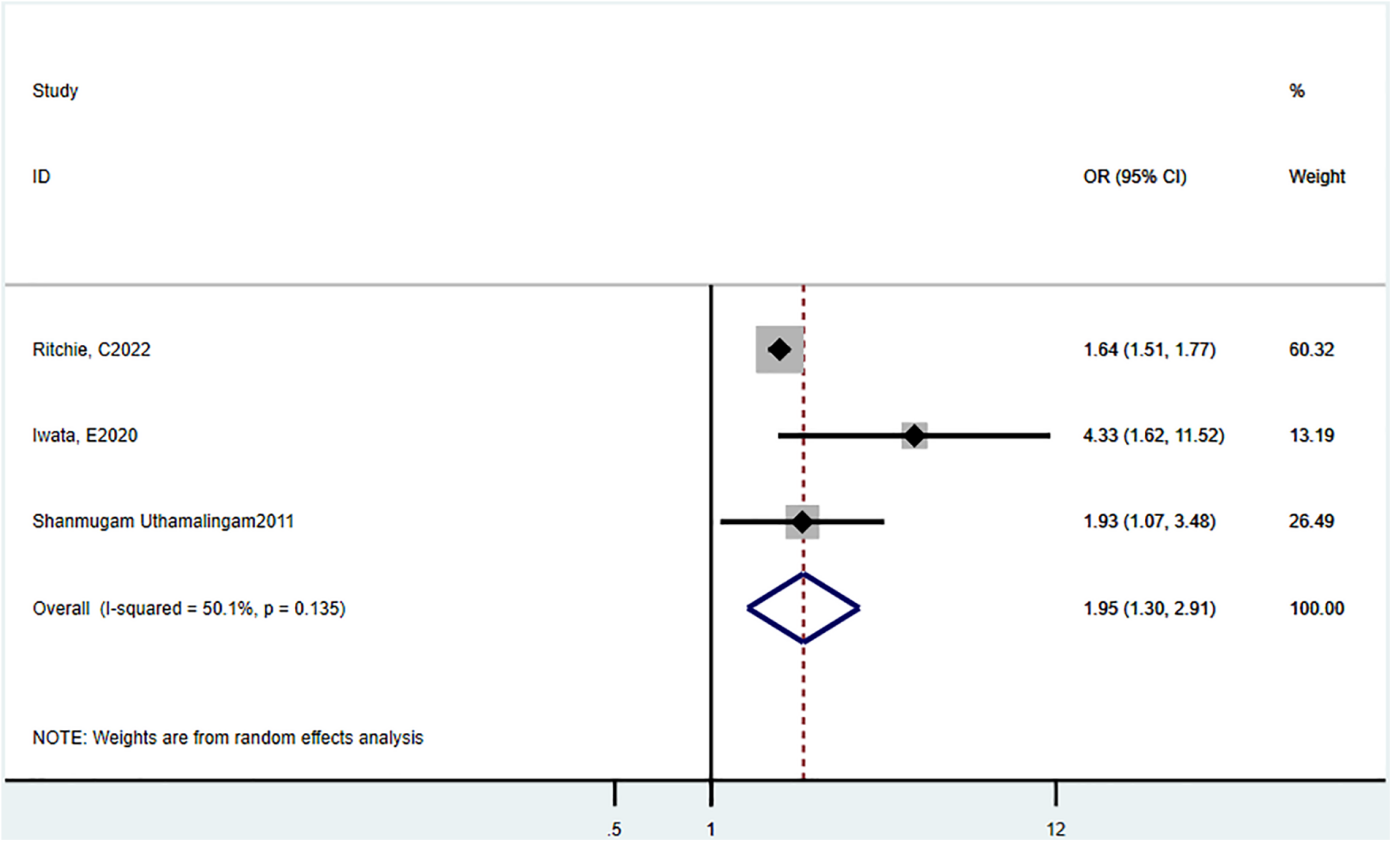
Odds ratio estimates of the effect of delirium comorbidity on in-hospital mortality of HF patients.

Three studies (16, 20, 22) reported in-hospital mortality. The estimated combined OR was 1.95 (95% CI = 1.30–2.91, *P *= 0.135, *I*^2^ = 50.1%) (Figure 2).

**Figure 3:**
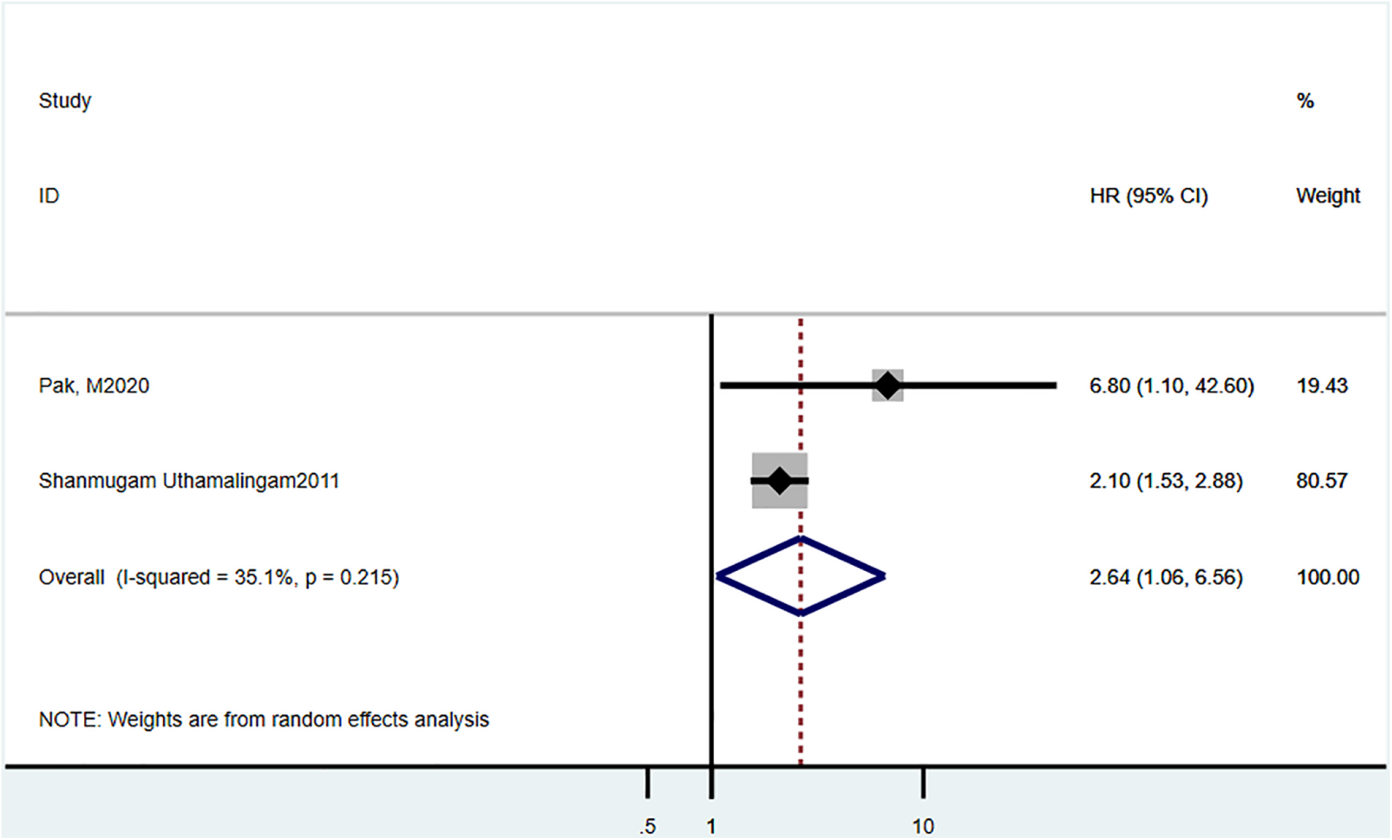
Hazard ratio estimates of the effect of delirium comorbidity on 90 day mortality of HF patients.

Two studies (19, 23) reported 90-day mortality, and the estimated pooled HR was 2.64 (95% CI = 1.06–1.56, *P *= 0.215, *I*^2^ = 35.1%) (Figure 3).

**Figure 4:**
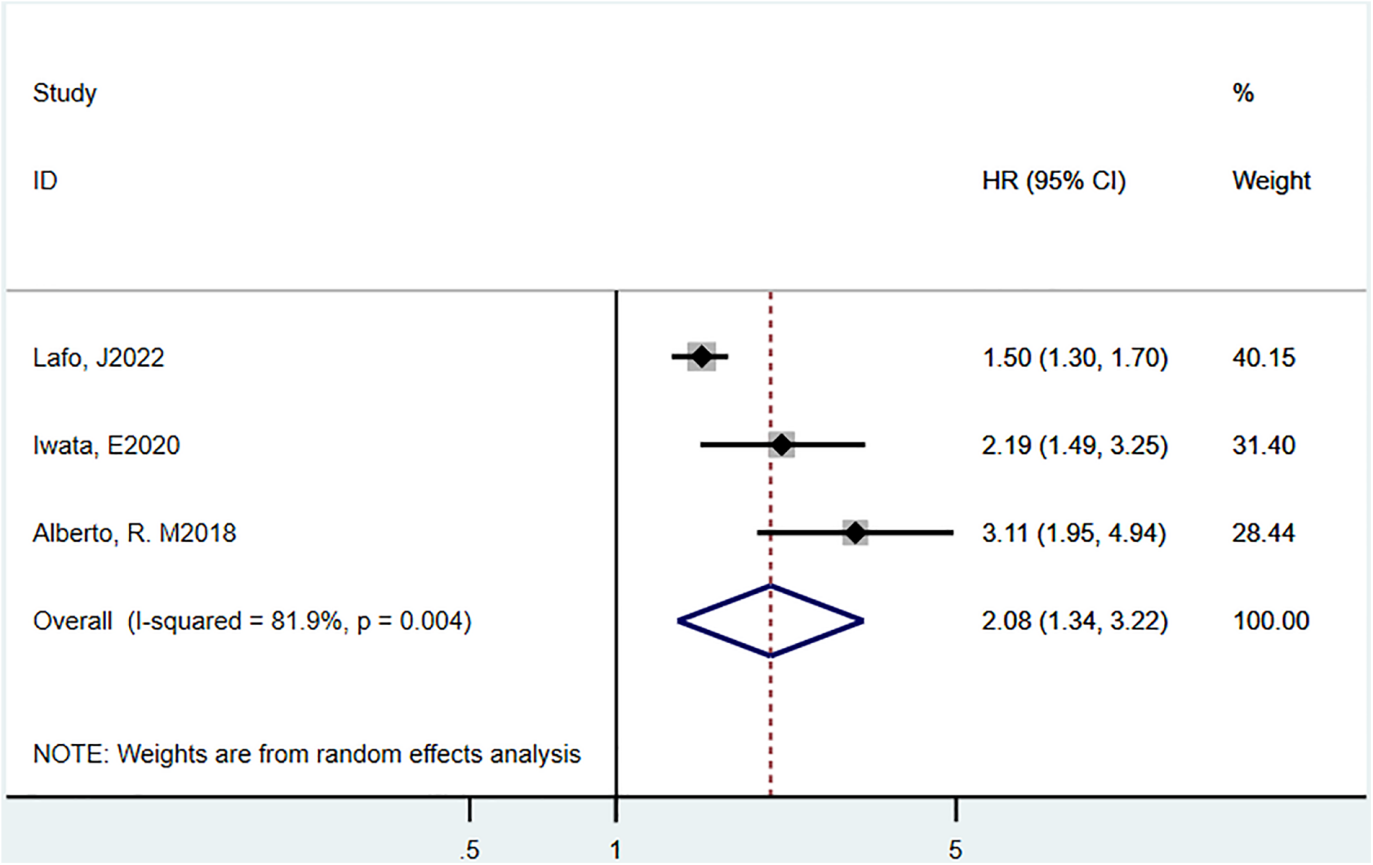
Hazard ratio estimates of the effect of delirium comorbidity on 365 day mortality of HF patients.

Three studies (17, 20, 22) reported 1-year mortality, and the estimated pooled HR was 2.08 (95% CI = 1.34–3.22, *P *= 0.004, *I*^2^ = 81.9%) (Figure 4).

### 3.5. Readmission rate

**Figure 5:**
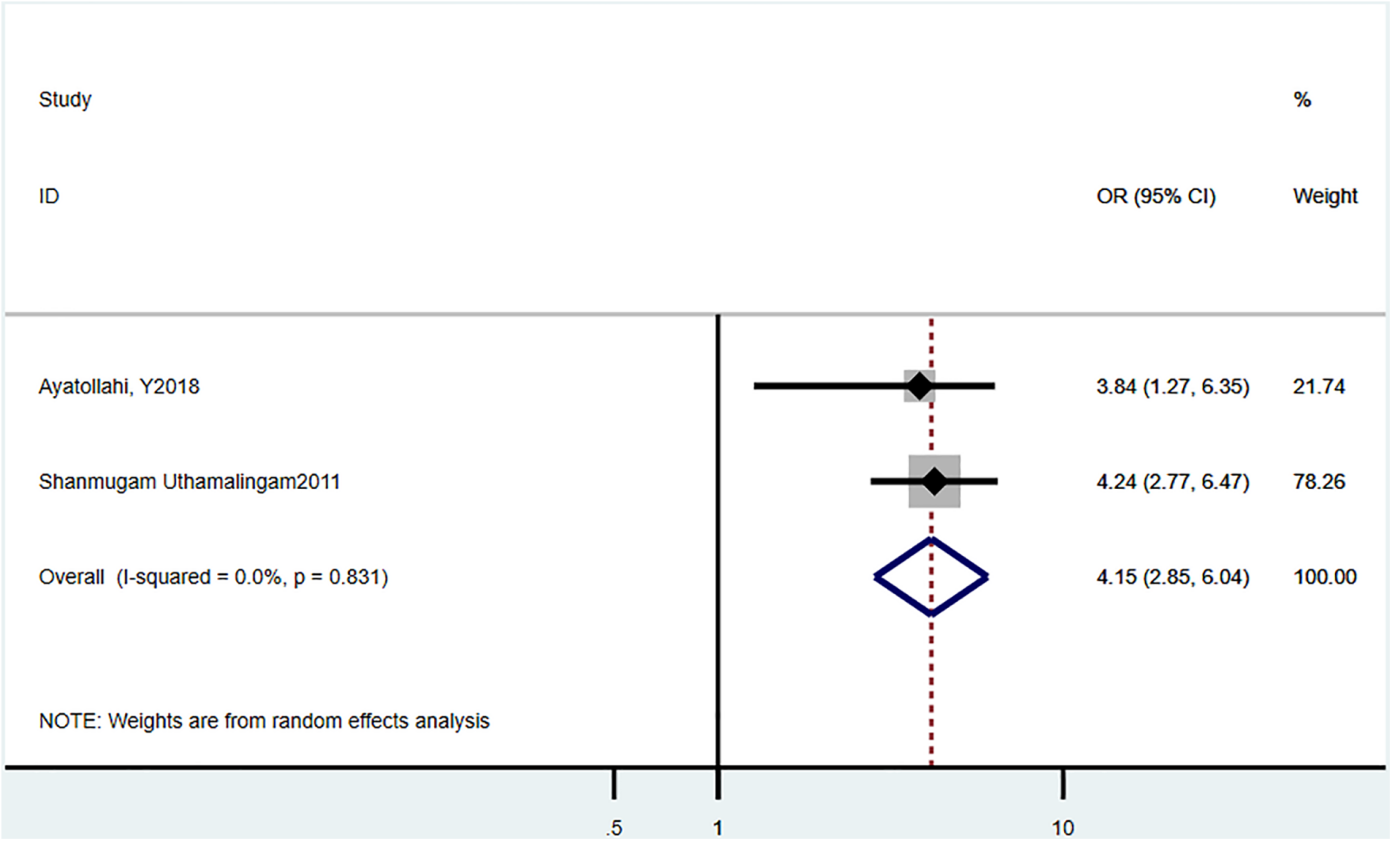
Estimates of the effect of HF comorbidity on 30 day readmission of HF patients.

Three studies (21, 23) reported the 30-day readmission rate, and the estimated pooled HR was 4.15 (95% CI = 2.85–6.04, *P *= 0.831, *I*^2^ = 0%) (Figure 5).

### 3.6. Subgroup analysis and sensitivity analysis

Subgroup analysis and sensitivity analysis were not conducted due to the limited number of studies.

## 4. Discussion

This is the first systematic review and meta-analysis that provided evidence for the impact of mental disorder on the prognosis of heart failure. Our results demonstrated that HF patients with delirium have significantly increased overall mortality, hospitalization time, and readmission rate.

HF is a complex clinical syndrome caused by reduced ventricular filling and ejection due to organic or functional dysfunction of the heart and hence the inability to meet the metabolic needs of the organs and tissues (33). HF has been shown to be an independent influencing factor for delirium (16–23).

Due to the inadequate impact of delirium on HF prognosis, medical workers were seldom trained on how to correctly communicate with HF patients with delirium. Fortunately, the diagnosis and clinical management of delirium in HF patients have gained increasing attention in recent years (10). The cause of the increased incidence of delirium in HF patients is currently unclear but is speculated to be associated with hemodynamic instability caused by HF. Hemodynamic instability can lead to cerebral hypoperfusion, which is believed to be related to the development of delirium (34, 35). Other causes may include HF-induced systemic inflammation (36), heightened sympathetic activity (37), and cardioembolic disease (38).

In this review, we analyzed the effect of delirium on all-cause 1-year mortality of HF patients reported in several studies. Lafo et al. (17) evaluated the 1-year all-cause mortality of 21,655 HF patients and found that those with delirium had significantly higher 1-year mortality than those without delirium (*P *< 0.001). The study by Lafo et al. was based on the data on veterans with heart failure admitted to skilled nursing facilities from the Veteran Administration. Similarly, a study by Pak et al. (20) based on 132 Japanese patients with acute decompensated heart failure admitted at Shimane University Hospital showed that patients with delirium had significantly higher incidences of mortality-related outcomes than those without delirium (21.6% vs. 3.9%, *P *= 0.002). Delirium was shown to be associated with higher mortality (adjusted HR = 6.8, 95% CI = 1.1–42.6, *P *= 0.042) (18), and hence early identification of patients at high risk of delirium may have significant prognostic implications. Consistent with these findings, our meta-analysis showed that HF patients with delirium had higher ORs for in-hospital mortality, 90-day mortality, and 1-year all-cause mortality than those without delirium.

We also assessed the risks of delirium-related readmission rate in HF patients. We found that numerous studies have reported an overall increase in hospitalization-related outcomes in HF patients with delirium. Lafo et al. reported that delirium was associated with the risk of readmission 30 days after discharge (18.6% patients, HR = 1.2, 95% CI = 1.0) (17). Uthamalingam et al. analyzed 883 consecutive patients with ADHF, >65 years, admitted at a large urban hospital, and demonstrated that delirium was an independent risk factor for 30-day readmission in HF patients (OR = 4.24, 95% CI = 2.77–6.47, *P *< 0.001) (23). In line with these findings, our meta-analysis showed that HF patients with delirium had higher HR for a 30-day readmission. Furthermore, the study by Ritchie et al. revealed that HF patients with delirium had prolonged hospitalization time than those without delirium (rate ratio = 1.47, 95% CI = 1.45–1.51) (17).Their study was based on adults with a primary diagnosis of HF in the National (Nationwide) Inpatient Sample database. However, due to insufficient numbers of studies reporting the association between length of hospital stay and delirium, we were unable to perform corresponding meta-analysis.

There are several limitations in this study. First, despite a comprehensive search, only a few eligible studies were included. As a result, we were unable to assess heterogeneity and bias, which may impact our results. Meanwhile, due to the limited number of original studies, subgroup analysis by HR or OR cannot be conducted to provide more detailed results. In addition, since the method for delirium assessment was not consistent among the included studies, we could not quantify the correlation between delirium severity and the prognosis and hospitalization-related outcomes of HF patients.

## 5. Conclusions

In summary, our study showed that delirium is associated with increased mortality and hospitalization-related risks in HF patients. The findings of our study may further improve the clinical understanding of delirium prognosis in HF patients and provide insights to the development of diagnosis and management guidelines, clinical pathways, and education for delirium in HF.

## Data Availability

The original contributions presented in the study are included in the article/Supplementary Material, further inquiries can be directed to the corresponding author.

## References

[1] Heidenreich PA Bozkurt B Aguilar D Allen LA Byun JJ Colvin MM 2022 AHA/ACC/HFSA guideline for the management of heart failure: a report of the American College of Cardiology/American Heart Association Joint Committee on clinical practice guidelines. Circulation. (2022) 145(18):e895–e1032. 10.1161/CIR.0000000000001062 35363499

[2] Kabbani S Al Habeeb W Liew HB Mohan JC Ogola E Sim D Supporting the management of patients with heart failure within Asia-pacific, Middle East, and African countries: a toolbox for healthcare providers. Cardiology. (2019) 142(Suppl 1):1–10. 10.1159/000496663 30947179

[3] Ni H Xu J. Recent trends in heart failure-related mortality: United States, 2000–2014. NCHS Data Brief. (2015) (231):1–8. PMID: 26727546

[4] GBD 2017 Disease and Injury Incidence and Prevalence Collaborators. Global, regional, and national incidence, prevalence, and years lived with disability for 354 diseases and injuries for 195 countries and territories, 1990–2017: a systematic analysis for the global burden of disease study 2017. Lancet. (2018) 392(10159):1789–858. 10.1016/S0140-6736(18)32279-7 30496104 PMC6227754

[5] Wong CY Chaudhry SI Desai MM Krumholz HM. Trends in comorbidity, disability, and polypharmacy in heart failure. Am J Med. (2011) 124(2):136–43. 10.1016/j.amjmed.2010.08.017 21295193 PMC3237399

[6] American Psychiatric Association A, Association AP. Diagnostic and statistical manual of mental disorders: DSM-5. Washington, DC: American Psychiatric Association (2013).

[7] Inouye SK. Delirium in older persons. N Engl J Med. (2006) 354(11):1157–65. 10.1056/NEJMra052321 16540616

[8] Wilson JE Mart MF Cunningham C Shehabi Y Girard TD MacLullich AMJ Delirium. Nat Rev Dis Primers. (2020) 6(1):90. 10.1038/s41572-020-00223-4 33184265 PMC9012267

[9] Marcantonio ER Ngo LH O’Connor M Jones RN Crane PK Metzger ED 3D-CAM: derivation and validation of a 3-minute diagnostic interview for CAM-defined delirium: a cross-sectional diagnostic test study. Ann Intern Med. (2014) 161(8):554–61. 10.7326/M14-0865 25329203 PMC4319978

[10] Barron EA Holmes J. Delirium within the emergency care setting, occurrence and detection: a systematic review. Emerg Med J. (2013) 30(4):263–8. 10.1136/emermed-2011-200586 22833596

[11] Gross AL Jones RN Habtemariam DA Fong TG Tommet D Quach L Delirium and long-term cognitive trajectory among persons with dementia. Arch Intern Med. (2012) 172(17):1324–31. 10.1001/archinternmed.2012.3203 23403619 PMC3740440

[12] Silver G Traube C Gerber LM Sun X Kearney J Patel A Pediatric delirium and associated risk factors: a single-center prospective observational study. Pediatr Crit Care Med. (2015) 16(4):303–9. 10.1097/PCC.0000000000000356 25647240 PMC5031497

[13] Velayati A Vahdat Shariatpanahi M Shahbazi E Vahdat Shariatpanahi Z. Association between preoperative nutritional status and postoperative delirium in individuals with coronary artery bypass graft surgery: a prospective cohort study. Nutrition. (2019) 66:227–32. 10.1016/j.nut.2019.06.006 31357095

[14] Sanford AM Flaherty JH. Do nutrients play a role in delirium? Curr Opin Clin Nutr Metab Care. (2014) 17(1):45–50. 10.1097/MCO.0000000000000022 24296414

[15] Smith TO Cooper A Peryer G Griffiths R Fox C Cross J. Factors predicting incidence of post-operative delirium in older people following hip fracture surgery: a systematic review and meta-analysis. Int J Geriatr Psychiatry. (2017) 32(4):386–96. 10.1002/gps.4655 28093812

[16] Ritchie C Walters RW Ramaswamy S Alla VM. Impact of delirium on mortality in patients hospitalized for heart failure. Int J Psychiatr Med. (2022) 57(3):212–25. 10.1177/00912174211028019 34176306

[17] Lafo J Singh M Jiang L Correia S Madrigal C Clements R Outcomes in heart failure patients discharged to skilled nursing facilities with delirium. ESC Heart Fail. (2022) 9(3):1891–900. 10.1002/ehf2.13895 35293145 PMC9065834

[18] Kwak MJ Avritscher E Holmes HM Jantea R Flores R Rianon N Delirium among hospitalized older adults with acute heart failure exacerbation. J Card Fail. (2021) 27(4):453–9. 10.1016/j.cardfail.2020.12.007 33347994

[19] Pak M Hara M Miura S Furuya M Tamaki M Okada T Delirium is associated with high mortality in older adult patients with acute decompensated heart failure. BMC Geriatr. (2020) 20(1):524. 10.1186/s12877-020-01928-7 33272204 PMC7713169

[20] Iwata E Kondo T Kato T Okumura T Nishiyama I Kazama S Prognostic value of delirium in patients with acute heart failure in the intensive care unit. Can J Cardiol. (2020) 36(10):1649–57. 10.1016/j.cjca.2020.01.006 32615071

[21] Ayatollahi Y Liu X Namazi A Jaradat M Yamashita T Shen JJ Early readmission risk identification for hospitalized older adults with decompensated heart failure. Res Gerontol Nurs. (2018) 11(4):190–7. 10.3928/19404921-20180322-01 29634848

[22] Alberto RM Domingo R Aitor A Sergio HM Pascual P Mireia P Long-term prognostic value of functional status and delirium in emergency patients with decompensated heart failure. Eur Geriatr Med. (2018) 9(4):515–22. 10.1007/s41999-018-0072-0 34674495

[23] Uthamalingam S Gurm GS Daley M Flynn J Capodilupo R. Usefulness of acute delirium as a predictor of adverse outcomes in patients >65 years of age with acute decompensated heart failure. Am J Cardiol. (2011) 108(3):402–8. 10.1016/j.amjcard.2011.03.059 21757045

[24] Hutt E Frederickson E Ecord M Kramer AM. Associations among processes and outcomes of care for medicare nursing home residents with acute heart failure. J Am Med Dir Assoc. (2003) 4(4):195–9. 10.1016/S1525-8610(04)70345-X 12837140

[25] Dungen HD Petroni R Correale M Coiro S Monitillo F Triggiani M A new educational program in heart failure drug development: the Brescia international master program. J Cardiovas Med (Hagerstown). (2018) 19(8):411–21. 10.2459/JCM.0000000000000669 29952846

[26] Page MJ McKenzie JE Bossuyt PM Boutron I Hoffmann TC Mulrow CD The PRISMA 2020 statement: an updated guideline for reporting systematic reviews. J Clin Epidemiol. (2021) 134:178–89. 10.1016/j.jclinepi.2021.03.001 33789819

[27] PROSPERO. International prospective register of systematic reviews. https://wwwcrdyorkacuk/PROSPERO/ (Accessed August 8, 2022).

[28] Stang A. Critical evaluation of the Newcastle-Ottawa scale for the assessment of the quality of nonrandomized studies in meta-analyses. Eur J Epidemiol. (2010) 25(9):603–5. 10.1007/s10654-010-9491-z 20652370

[29] Higgins JP Thompson SG Deeks JJ Altman DG. Measuring inconsistency in meta-analyses. Br Med J. (2003) 327 (7414):557–60. 10.1136/bmj.327.7414.557 12958120 PMC192859

[30] Correale M Altamura M Carnevale R Tricarico L Malerba S Gallotta AM Delirium in heart failure. Heart Fail Rev. (2020) 25(5):713–23. 10.1007/s10741-019-09842-w 31377979

[31] Smith MJ Breitbart WS Platt MM. A critique of instruments and methods to detect, diagnose, and rate delirium. J Pain Symptom Manage. (1995) 10(1):35–77. 10.1016/0885-3924(94)00066-T 7714346

[32] Ely EW Margolin R Francis J May L Truman B Dittus R Evaluation of delirium in critically ill patients: validation of the confusion assessment method for the intensive care unit (CAM-ICU). Crit Care Med. (2001) 29(7):1370–9. 10.1097/00003246-200107000-00012 11445689

[33] McDonagh TA Metra M Adamo M Gardner RS Baumbach A Böhm M 2021 ESC guidelines for the diagnosis and treatment of acute and chronic heart failure. Eur Heart J. (2021) 42(36):3599–726. 10.1093/eurheartj/ehab368 34447992

[34] Fong TG Bogardus ST Jr. Daftary A Auerbach E Blumenfeld H Modur S Cerebral perfusion changes in older delirious patients using 99mTc HMPAO SPECT. J Gerontol A Biol Sci Med Sci. (2006) 61(12):1294–9. 10.1093/gerona/61.12.1294 17234823

[35] Yokota H Ogawa S Kurokawa A Yamamoto Y. Regional cerebral blood flow in delirium patients. Psychiatry Clin Neurosci. (2003) 57(3):337–9. 10.1046/j.1440-1819.2003.01126.x 12753576

[36] Athilingam P Moynihan J Chen L D'Aoust R Groer M Kip K. Elevated levels of interleukin 6 and C-reactive protein associated with cognitive impairment in heart failure. Congest Heart Fail. (2013) 19(2):92–8. 10.1111/chf.12007 23057677 PMC3801169

[37] Honda S Nagai T Sugano Y Okada A Asaumi Y Aiba T Prevalence, determinants, and prognostic significance of delirium in patients with acute heart failure. Int J Cardiol. (2016) 222:521–7. 10.1016/j.ijcard.2016.07.236 27509220

[38] de la Torre JC. Cardiovascular risk factors promote brain hypoperfusion leading to cognitive decline and dementia. Cardiovasc Psychiatry Neurol. (2012) 2012:367516. 10.1155/2012/367516 23243502 PMC3518077

